# Gun-Shot Wound of the Neck—Injury of the Spinal Cord

**Published:** 1867-09

**Authors:** Stith Malone

**Affiliations:** Athens, Ala.


### ARTICLE II.

### *Gun-shot Wound of the Neck—Injury of the Spinal Cord.*

By Stith Malone, A. M., M. D., of Athens, Ala.

James D. Grubs, a member of a Tennessee Cavalry Regiment, was wounded in the town of Athens, Ala., on the 24th September, 1864, in a battle fought between the Confederates, under General Forrest, and the U. S. forces—81st Ohio Regiment—under Lieut. Col. Elliot, sent from Decatur to the assistance of the negro garrison, stationed here, under Col. Campbell. Grubs was found the morning after the battle, with "his neck broke." He was carried to the hospital, some quarter of a mile, where he remained a few days, the surgeons expecting that each day would be his last; but continuing to live, he was again moved about the same distance, to secure better attention. He remained in this family three weeks, when it became necessary to move him again, about the same distance.

I saw Grubs, for the first time, on the fifth day after he received his wound. I found him suffering greatly, complaining of his neck and bowels; the latter being *extremely* painful; his pulse 142; his extremities cool, very much so—and excessively restless. On examining his wound, I found a ball had passed through his neck, and as well as I could ascertain, had cut across the spinal marrow, perhaps the posterior half of it. Grubs was managed as well as we could under the circumstances. He lived fifty-one days, and finally succumbed, it seemed to me, from dysinteric symptoms, attended with extreme and persistent nausea. His bowels, at times, gave him so much pain, before the development of the dysinteric symptoms, that spasms and tetanus were thought to be imminent. Voluntary motion was almost entirely lost; he had a little use of his left hand. His evacuations were only partially under his control, but never required the use of an instrument for their withdrawal.

On the 51st day he died, and in four hours an examination was made of his neck, with the following result: The ball had passed through the lamina of the fourth cervical vertebra, dissevering entirely the laminal attached to the vertebra from the spinal portions, so that the spinal portions of the bone were lying loose in the half formed sack around the injured bone. The sheath of the spinal marrow, posteriorly, was cut across, and about 4-10ths of the spinal marrow itself, was severed. The internal wound, including the sheath of the cord, was filled with an offensive, saneous pus, tinged with blood.

